# The impact of screen exposure among school-aged children in South India during the COVID-19 pandemic: an online survey

**DOI:** 10.1186/s43163-023-00396-1

**Published:** 2023-02-23

**Authors:** Teja Deepak Dessai, Surakshya Sigdel, Thakendra Chand, Rashmi J. Bhat, Kaushlendra Kumar

**Affiliations:** 1 Bangalore Speech and Hearing Research Foundation, Bangalore-Manipal Academy of Higher Education, Manipal, Karnataka, India; 2 Dr. S. R. Chandrasekhar Institute of Speech and Hearing, Bangalore, India; 3 Department of Audiology and Speech Language Pathology, Kasturba Medical College, Mangalore, Manipal Academy of Higher Education, Manipal, Karnataka, India

**Keywords:** Screen time, Personal Listening Devices, Vision, Balance, Hearing

## Abstract

**Background:**

The impact of excessive screen time with personal listening devices (PLDs) on vision, hearing, balance, and overall health among adults has been reported in the literature. However, its impact on children is not well documented. A survey was undertaken to highlight the possible effects of screen time combined with transducers on vision, hearing, balance, and overall health complaints in children via parental proxy. This cross-sectional survey consisted of questions divided into four domains: vision, hearing, balance, and overall health. It was conducted online using social media to avail total maximum responses.

**Results:**

A total of 136 responses were obtained from the parents of children studying from grade 1 to 8 through the survey conducted in south India. Increased negative impact on vision was observed in more than 50% of children. Similarly, the hearing and balance domain reported 16% ear pain, 4.4% tinnitus, 9.6% dizziness, 8.8% nausea, and 2.2% imbalance while walking. Likewise, the overall stress domain reported 26.5% general body discomfort, 37.5% neck pain and stiffness, 29.4% headaches, 43.4% lack of concentration, 39.7% overall changes in child’s health and 46.3% behavioral issues with various severity markings (slight to severe).

**Conclusions:**

Longer periods of screen exposure have various negative impacts on vision, hearing, balance system, and overall health. These effects have to be managed for the health of our children.

**Supplementary Information:**

The online version contains supplementary material available at 10.1186/s43163-023-00396-1.

## Background

With the COVID-19 pandemic across the globe, an unprecedented increase in screen time is observed. The prevention measures including lockdowns, along with strict containment measures, led to school closures and an increase in the use of electronic equipment in families to educate, entertain, and connect their children to the outside world.

Considering the rapid expansion of technology and the immense need to keep children occupied, gadgets are often given to children as gifts. With an onslaught of the global pandemic, this need has further grown to include the gadget and its screen time to participate in online classes through schools. Screen time paired with its accessories such as headphones or earphones has a detrimental effect on the human system. Children who use screen time for 2 or more hours per day score lower in language and thinking tests. Evidence of eye strain, eye irritation, burning sensations in the eyes, redness of the eyes, and blurred vision among adults is also reported by Bergqvist and Knave (1994) [1].

Unlike other parts of the body, damage to the inner ear is irreversible, and with prolonged exposure, the damage can become even worse, causing permanent hearing loss. Children in the USA who were using personal listening devices to listen to music were reported with a 12.5% hearing loss caused by music (2017) [2].

An individual’s vision, hearing, balance and overall health can be negatively impacted by prolonged screen time. However, the effects of longer screen time in combination with earphones or headphones on vision, hearing, balance, and overall health is not well documented among children. Hence, the survey intended to uncover any complaints parents might have related to vision, hearing, balance and general health.

### Aim of the study

To highlight the possible effects of longer screen time with/without earphones or headphones on vision, hearing, balance and overall health.

## Method

The study was carried out after obtaining the Dr. S. R. Chandrasekhar Institute of Speech and Hearing Institute Ethical Committee’s approval. Respondents gave their written consent to participate in the study by clicking a response button on the first page of the questionnaire website, which offered information about informed written consent. The survey consisted of 43 questions covering areas such as family and gadget use, vision, hearing, balance, and overall health. An assortment of multiple-choice questions was prepared. All questions were reviewed and content validated by professionals with at least 5 years of experience in the respective field. The formulated survey was first used to carry out a pilot study in order to understand and tackle the difficulties one may face while answering the survey.

The study examined the responses of parents who had children between the ages of 6–12 years attending online classes during the COVID-19 pandemic and residing in Southern India. Prior to analysis, responses obtained from parents of disabled children who did not belong to the targeted age or regional group were excluded. This online survey was conducted in the English language. All parents who could read and write in English could participate in this online survey. The survey was conducted for a duration of 1 year from 2020–2021.

In order to make this survey widely accessible, Google Forms (Google Inc., Mountain View, CA, USA) were used. It was forwarded through social network forums (WhatsApp & Messenger) so that all parents of children attending online classes in Southern India could participate. The survey was also circulated to the parents via electronic mail. We chose an online survey as a cost-effective and time-saving means of reaching a large number of people in Southern India.

## Results

The study analyzed the responses of 136 parents of children attending online classes during the pandemic COVID-19 using frequency tables. The participants answered all the questions from the four domains.

### General details of the family and online classes

The study’s participants were parents of children aged 6 to 12 years (mean age of 9.22 years) (Table 1).

**Table 1:** Total number of children and class grades attended through online mode

Class grade attending to through online mode	Counts	Percentage (%) of total no. of children	Cumulative percentage (%)
1	19	14.0	14.0
2	31	22.8	36.8
3	14	10.3	47.1
4	11	8.1	55.1
5	9	6.6	61.8
6	23	16.9	78.7
7	24	17.6	96.3
8	5	3.6	100

The majority of the study participants were second graders followed by the seventh- and sixth-grade children (Table 2).

**Table 2:** Frequency of total number of children in the house

Total number of children in the house	Counts	Percentage (%) of total children in the house	Cumulative percentage (%)
1	43	31.6	31.6
2	76	55.9	87.5
3	11	8.1	95.6
4	5	3.7	99.3
More than 4	1	0.7	100

The number of children in the home as a whole was one of the survey’s questions. Only 0.7% of parents said they had more than four children, while 31.6% said they only had one child.

Questions were posed to elicit responses that would demonstrate parent's understanding of the impact of online classes. The computer vision syndrome was recognized by 41.9% of the parents. Every parent (100%) had to enroll their children in online classes. More information about the child’s online classes can be found below in Table 3.

**Table 3:** Data on Gadget use

Questions	Options	Frequency (percentages)
Are you aware of computer vision syndrome?	No	57 (41.9%)
	Yes	79 (58.1%)
Does your child attend online classes?	Yes	136 (100%)
Are your child’s online classes continuous?	No	74 (54.4%)
	Yes	62 (45.6%)
Does your child get break in between online classes?	No	10 (7.4%)
	Yes	126 (92.6%)
If yes, what is the duration of break your child gets?	<15 min	47 (36.2%)
	15–20 min	46 (35.4%)
	>20 min	37 (28.5%)
Does your child attend online classes using gadgets such as laptop, computer, tablet/iPad, mobile phone?	Yes	136 (100%)
What is the total duration of online classes per day?	<2 h	48 (35.3%)
	2–4 h	60 (44.1%)
	4–6 h	23 (16.9%)
	> 6 h	5 (3.7%)
Have you allocated a separate gadget for your child’s use only?	No	48 (35.3%)
	Yes	88 (64.7%)
Does your child use the above mentioned gadgets apart from the online classes?	No	40 (29.4%)
	Yes	96 (70.6%)
What is the accessory used by your child for online classes?	Earphone	15 (11.0%)
	Headphone	32 (23.5%)
	Speaker	89 (65.4%)
What is the volume level used on the gadgets during online classes?	<30%	17 (12.5%)
	30–40%	33 (24.3%)
	40–60%	58 (42.6%)
	>60%	28 (20.6%)

The respondents were asked to indicate the length of time their children spent in online classes, the use of gadgets and the volume levels they utilized on their gadgets, and the accessories they used for attending online classes as well as leisure activities.

### Vision

As a variety of elements have shown to influence vision, questions on vision have been included in this category. Respondents were asked about the techniques they used with their child while taking online classes as part of the study to gauge how necessary it is to take precautions while attending online classes. In the vision domain, Table 4 shows the responses of parents.

**Table 4:** Responses of the vision domain

Questions	Options	Frequency (percentages)
Does the gadget used by your child have anti-glare screen?	No	85 (62.5%)
	Yes	51 (37.5%)
Does your child use anti-glare glasses while using gadget?	No	105 (77.2%)
	Yes	31 (22.8%)
Does your child adjust brightness level on gadget during use?	No	62 (45.6%)
	Yes	74 (54.4%)
What is the total distance maintained between your child and the gadget for online classes?	<20 inches	50 (36.8%)
	20-40 inches	81 (59.6%)
	>40 inches	5 (3.7%)
What is the angle maintained between your child and gadget for online classes?	<15 degree	34 (25.0%)
	15-20 degree	82 (60.3%)
	>20 degree	20 (15.7%)
Does your child complain of eye strain or pain during or after?	No	102 (75%)
	Yes	34 (25%)
If yes, what is the severity of eye strain or pain?	Slight	23 (67.6%)
	Moderate	10 (29.4%)
	Severe	1 (2.9%)
Does your child complain of blurred vision during or after online classes?	No	117 (86.02%)
	Yes	19 (13.9%)
What is the severity of blurred vision experienced by your child?	Slight	16 (84.2%)
	Moderate	3 (15.8%)
	Severe	0 (0%)

The execution of tactics for internet visibility was cited by nearly half of the respondents. However, as evidenced by the data, some youngsters complained of eyesight issues.

### Hearing and Balance 

According to the findings of the current study, students who took online classes experienced ear-related symptoms in varying severities, such as ear discomfort (11.8%), tinnitus in the right ear only (0.7%), in the left ear only (1.5%), in both ears (2.2%), nausea (8.08%), dizziness (9.6%), and imbalance when walking (2.2 %) (Table 5).

**Table 5:** Responses to the hearing and balance domains

Questions	Options	Frequency (percentages)
Does your child complain of ear pain or discomfort during or after online classes?	No	120 (88.2%)
	Yes	16 (11.8%)
If yes, what is the severity of ear pain or discomfort experienced by your child?	Slight	10 (62.5%)
	Moderate	4 (25%)
	Severe	2 (12.5%)
Does your child complain of ringing sensation in ears?	No	130 (95.6%)
	Yes, in right ear	1 (0.7%)
	Yes, in both ear	3 (2.2%)
	Yes, in left ear	2 (1.5%)
If yes, what is the sensitivity of ringing sensation in ears?	Slight	0 (0%)
	Moderate	1 (16.7%)
	Severe	5 (83.3%)
Does your child complain of nausea and/ or sweating during or post online classes?	No	125 (91.9%)
	Yes	11 (8.08%)
If yes, what is the severity of nausea and sweating experienced by your child?	Slight	8 (72.7%)
	Moderate	3 (27.3%)
	Severe	0 (0%)
Does your child complain of dizziness during or after online class?	No	123 (90.4%)
	Yes	13 (9.6%)
If yes, what is the severity of dizziness experienced by your child?	Slight	10 (76.9%)
	Moderate	3 (23.1%)
	Severe	0 (0%)
Does your child complain about imbalance while walking?	No	133 (97.8%)
	Yes	3 (2.2%)

### Overall health

Finally, the fourth domain focused on topics pertaining to the overall health of children enrolled in online classes as a result of the pandemic. The questions were about general health and the responses revealed a set of symptoms that the children had after attending online lessons. General bodily discomfort (25.7%), neck pain and stiffness (35.2%), headaches (28.6%), lack of attention (43.4%), overall changes in child's health (38.9%) and behavioral concerns (46.3%) were also noted by respondents with varying degrees of severity. In addition, a question was posed to determine the overall stress experienced by parents whose children were enrolled in online programs as a result of the pandemic, and 58.1% of parents said they were stressed (Table 6).

**Table 6:** Responses from the overall health domain

Questions	Options	Frequency (percentages)
Does your child complain of general body discomfort?	No	101 (74.2%)
	Yes	35 (25.7%)
If yes, what is the severity of discomfort experienced by your child?	Slight	21 (60.0%)
	Moderate	10 (28.6%)
	Severe	4 (11.4%)
Does your child complain neck pain and stiffness during or after online class?	No	88 (64.7%)
	Yes	48 (35.2%)
If yes, what is the severity of neck pain and stiffness experienced by your child?	Slight	30 (62.5%)
	Moderate	10 (20.8%)
	Severe	8 (16.7%)
Does your child complain of headache and/ or heavy headedness?	No	97 (71.3%)
	Yes	39 (28.6%)
If yes, severity of headache or heavy headedness experienced by your child?	Slight	24 (61.5%)
	Moderate	12 (30.8%)
	Severe	3 (7.7%)
Does your child experience difficulty concentrating or focusing lately?	No	77 (56.6%)
	Yes	59 (43.4%)
If yes, what is the severity of difficulty experienced in focusing lately?	Slight	34 (59.6%)
	Moderate	20 (35.1%)
	Severe	3 (5.3%)
Have you noticed overall change in your child’s health during COVID?	No	83 (61.02%)
	Yes	53 (38.9%)
If yes, what is the severity of change in health condition of your child?	Slight	30 (56.6%)
	Moderate	20 (37.7%)
	Severe	3 (5.7%)
Have you observed any behavioral issues?	No	73 (53.7%)
	Yes	63 (46.3%)
If yes, what are the behavioral issues observed?	Hyperactivity	9 (14.3%)
	Inattentive	22 (34.9%)
	Irritability	32 (50.8%)
Have you observed overall stress in your routine during this covid-19 pandemic?	No	57 (41.9%)
	Yes	79 (58.1%)
If yes, what is the severity of stress experienced by you?	Slight	28 (35.4%)
	Moderate	35 (44.3%)
	Severe	16 (20.3%)

## Discussion

The study aimed to highlight the possible effects of longer screen time with/without earphones or headphones on vision, hearing, balance, and overall health. A total of 136 survey responses were analyzed to meet the aim of the study. All the domains considered in the survey showed a negative impact of longer screentime time on children.

In today’s technological and media-driven society, many parents utilize screens to amuse or divert their young children while juggling other requirements. From the results of the study, we can understand that 31.6% of the parents reported a single child in the family. Given that the pandemic restricted outdoor activities, leaving the youngster with a device was an option. This thereby might have contributed to longer screen times and its negative impacts on the assessed 4 domains. To add on, responses obtained from the survey highlighted children as young as 2nd grade to have experienced a negative impact of screen time. This was followed by seventh and sixth-grade children.

### Vision

Porcar, Pons, and Lorente (2016) [3] reported exposure to longer screen times is known to be associated with symptoms such as eyestrain, headaches, dry eyes, blurred vision, and neck and shoulder pain. These symptoms together are known as computer vision syndrome (CVS). Most computer users suffer from CVS, but with the requirement to attend online classes, even children are at a greater risk of developing it. Increased vision domain symptoms in youngsters could be due to a lack of awareness. Out of 136 parents, 57 (41.9%) had no awareness about CVS. As a result, symptoms relating to vision might have intensified. Similarly, the general population may be uninformed of CVS’ presence.

An increased harmful impact on eyesight is attributable to students spending more than 2–4 h per day on video display terminals, with just a 15-min break between classes (less than recommended), and a lower percentage of youngsters wearing anti-glare screens while using the gadgets (Shrestha, Mohamed, and Shah 2011) [4]. The minimum angle and distance between the user and the screen, according to the US Department of Labor Occupational Safety and Health Administration in 2022 [5], should be 20° below horizontal eye level and 20–40 in respectively. The participants in the current study, on the other hand, lacked this understanding, which may have elevated the overall risk of eyesight health. Appearance of eye pain or strain, as well as small and moderate impaired vision, can be ascribed to the usage of devices with incorrect brightness settings.

### Hearing and Balance

India is experiencing a significant increase in students taking online programs. Almost every youngster from this study owns and uses various gadgets for both school and leisure purposes paired with headphones or earphones, with most of them cranking up the volume to 40–60%. A comprehensive study conducted by the Centers for Disease Control and Prevention on 3116 young children aged 9–11 years old found a link between earphones and hearing loss in 2011–2012 [6]. In comparison to their peer group, 40% of study participants had difficulty hearing at higher frequencies. Furthermore, Kashyap and Bhatia (2018) [6] found that people who use personal devices for 1 to 3 h per day have greater behavioral hearing thresholds. In a study of young personal listening device users, Sulaiman, Husain, and Seluakumaran (2014) [7] reported early hearing impairment. The safe listening duration for personal listening devices combined with earphones, according to Portnuff, Fligor, and Arehart (2011) [8], is 4 h per day at 70% volume or 90 min per day at 80% volume. As a result, prolonged exposure to screens, as well as listening to longer periods at higher volume levels, may represent a lifelong harm to a child’s hearing, balance, and overall health.

Additionally, the presence of symptoms such as nausea, sweating after classes, imbalance, or vertigo in the current survey can be linked to the cochlear and vestibular systems sharing a common genesis [9–13]. It is a well-known fact that auditory overstimulation has a significant risk of damaging the hair cells of the cochlea and the afferent neurons that innervate them, resulting in temporary or permanent hearing loss. The vestibular system, like the cochlea, is contained in the temporal bone and membranous labyrinth of the inner ear. Both system's sensory hair cells have a similar shape and use the same VIII cranial nerve to translate hair cell bundle displacement into brain activity [14, 15]. Azizi (2010) [16] stated that semi-circular canals and the vestibular end organs are stimulated by the intercommunications between the fluids of the cochlea. As a result, any danger of injury to the hearing system may have an adverse effect on balance problems. It is well established from the data of this study that children utilize devices for online lessons and recreational purposes, exceeding the daily screen time limit while using earphones or headphones as 96% of the parents reported children’s usage of gadgets beyond class hours daily. The impact of increasing levels on gadgets for online classes during the COVID-19 epidemic for over a year may have caused an early impairment to the hearing and balance system.

The usage of headphones that are worn over or around the ear can cause pressure injury to the pinna, which is the outer part of the ear. Bending or squeezing the delicate pinna cartilage under headphones can cause pain and run the risk of causing a skin abrasion that could become infected, with the risk being higher for individuals who already had a chronic middle ear infection. Mazlan, Saim, Thomas, Said, and Liyab (2002) [17] reported that its use could cause increased itchiness and reactivation of the middle ear infection. Complaints of ear pain and discomfort (11.8%) and tinnitus (2.2%) in both ears were documented in the current investigation, with a severity of 83.3%.

### Overall stress

Long-term use of devices might have negative consequences on a person’s overall health. Literature reports several symptoms such as disorientation, apathy, fatigue, dizziness, headache, increased salivation, dry mouth, difficulty focusing, eye strain, vomiting, pallor, sweating, postural instability, and general discomfort [18, 19]. Symptoms of various severity (slight–severe) were observed in youngsters during and after online classes in the current investigation. Frequent or prolonged computer usage increases the risk of neck and shoulder pain, low back pain, and arthritis in fingers along with general body fatigue and discomfort [20]. Similar findings are obtained in the study where children have complained of general discomfort along with neck and shoulder stiffness majorly of slight to moderate degrees and few have reported with severe issues. Montagni, Guichard, Carpenet, Tzourio, and Kurth (2016) [21] quote that electronic devices give off high-energy, short-wavelength blue, and violet lights; high levels of screen time exposure were found to be associated with migraine in young adults. This study supports the findings of the survey. A total of 29.4% of children complained of headache and heavy headedness. An overall degradation in the health of a slight to severe degree has been reported by 39.7% of children post online classes and results suggest 51.1% parents of children attending online classes have felt the increase in the frequency of stress of a moderate degree post-COVID-19 pandemic.

Non-wandering mind, strong social functioning and attachment, and good physical health are all important components of psychophysiological resilience. Children and adolescent's excessive usage of digital media appears to be a primary factor impeding the development of sound psychophysiological resilience [22]. The observed behavioral alterations in 46.3% of children attending online classes, such as irritation, inattentiveness, and hyperactivity (shifting attention from one task to the other), could be the result of persistent disturbances in the parent's psychophysiological resilience.

The purpose of the study was to determine how screen time affected vision, hearing, balance and overall health among children. The poll also looked at how well parents of students who attend online classes are cognizant about the consequence of increased screen time due to the COVID-19 pandemic. The several domains emphasized the most prevalent symptoms that a youngster with a gadget can suffer from. These detrimental effects of increased screen time can have a long-term impact on the visual, hearing, balance and overall health. As a result, it is critical that the general population is educated about the dangers of excessive screen time.

Further research is essential to broaden the knowledge about the effects of screen time on vision, hearing, balance, and overall general health disclosure gained via this study. Despite the limitations of the study (low sample size and responses only through online mode which might have restricted the participation of illiterate parents), we feel that it is timely and appropriate in its present form as it is one of the primary studies to explore negative effects on the child and his/her sensory systems. Additional studies are warranted to identify the knowledge of the general public about the negative impacts of longer screen time usage. We also recommend further studies to explore and develop awareness strategies to reduce the increasing number of symptoms related to screen time.

## Conclusion

Four domains considered in the study highlighted the negative impact of screen time on vision, hearing, balance, and overall health of children as young as 1st graders. The practice of PLD usage if persists may lead to irreparable damage to the humans. Therefore, it is of immense importance that the parents are well educated about the adverse effects of longer screen time. Likewise, educating clinicians with this information will serve as a deciding factor in assessment and management thereby, improving the overall quality of life.

## Supplementary Information

**Additional file 1.**

## Data Availability

The datasets generated and/or analysed during the current study are available in the repository: https://urldefense.com/v3/, https://docs.google.com/spreadsheets/d/1FhDFzSxkqbQ-Abz__3tirdhmw3Kj8RiF/edit?usp=sharing&ouid=117658435603478772767&rtpof=true&sd=true__;!!NLFGqXoFfo8MMQ!qP_fOmJdzJ9UVqu8711froKMwJkrN4pDdDo6UXGN449NquJVQaJ3AfP2o2QlFhtLBucKeZ4A7bQ2c-oJzRtl5_P7uwAo$. All data generated or analysed during this study are included in this published article (and its Supplementary information files).
